# Dibromidotris(dimethyl­amine)magnesium(II)

**DOI:** 10.1107/S1600536809024702

**Published:** 2009-07-08

**Authors:** Hannes Vitze, Hans-Wolfram Lerner, Michael Bolte

**Affiliations:** a Institut für Anorganische Chemie, J. W. Goethe-Universität Frankfurt, Max-von-Laue-Strasse 7, 60438 Frankfurt/Main, Germany

## Abstract

The Mg centre in the title compound, [MgBr_2_(C_2_H_7_N)_3_], is penta­coordinated in a trigonal-bipyramidal mode with the two Br atoms in axial positions and the N atoms of the dimethyl­amine ligands in equatorial positions. The Mg^II^ centre is located on a crystallographic twofold rotation axis. The crystal structure is stabilized by N—H⋯Br hydrogen bonds. The N atom and H atoms of one dimethylamine ligand are disordered over two equally occupied positions.

## Related literature

The solid-state structures of Mg–Br compounds feature coordination numbers of the Mg center from four as in [MgBr(Si^*t*^Bu_3_)(THF)]_2_ (Lerner *et al.*, 2003 ▶) to six as in [MgBr_2_(THF)_4_] (Lorbach *et al.*, 2007 ▶).
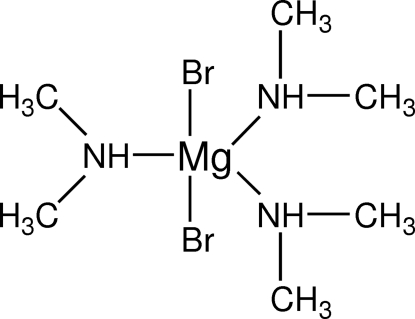


## Experimental

### 

#### Crystal data

[MgBr_2_(C_2_H_7_N)_3_]
*M* *_r_* = 319.39
Hexagonal, 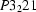

*a* = 9.0951 (7) Å
*c* = 14.4544 (12) Å
*V* = 1035.49 (14) Å^3^
*Z* = 3
Mo *K*α radiation
μ = 5.88 mm^−1^
*T* = 173 K
0.25 × 0.25 × 0.23 mm

#### Data collection

Stoe IPDSII two-circle diffractometer
Absorption correction: multi-scan (*MULABS*; Spek, 2003 ▶; Blessing, 1995 ▶) *T*_min_ = 0.321, *T*_max_ = 0.345 (expected range = 0.241–0.258)
5279 measured reflections
1285 independent reflections
1223 reflections with *I* > 2σ(*I*)
*R*_int_ = 0.037

#### Refinement

*R*[*F* ^2^ > 2σ(*F* ^2^)] = 0.022
*wR*(*F* ^2^) = 0.049
*S* = 0.96
1285 reflections
61 parameters
H-atom parameters constrained
Δρ_max_ = 0.25 e Å^−3^
Δρ_min_ = −0.34 e Å^−3^
Absolute structure: Flack (1983 ▶), 517 Friedel pairs
Flack parameter: −0.012 (17)

### 

Data collection: *X-AREA* (Stoe & Cie, 2001 ▶); cell refinement: *X-AREA*; data reduction: *X-AREA*; program(s) used to solve structure: *SHELXS97* (Sheldrick, 2008 ▶); program(s) used to refine structure: *SHELXL97* (Sheldrick, 2008 ▶); molecular graphics: *XP* in *SHELXTL-Plus* (Sheldrick, 2008 ▶); software used to prepare material for publication: *SHELXL97*.

## Supplementary Material

Crystal structure: contains datablocks I, global. DOI: 10.1107/S1600536809024702/at2830sup1.cif

Structure factors: contains datablocks I. DOI: 10.1107/S1600536809024702/at2830Isup2.hkl

Additional supplementary materials:  crystallographic information; 3D view; checkCIF report

## Figures and Tables

**Table 1:** Hydrogen-bond geometry (Å, °)

*D*—H⋯*A*	*D*—H	H⋯*A*	*D*⋯*A*	*D*—H⋯*A*
N1—H1⋯Br1^i^	0.93	2.90	3.638 (2)	137
N2—H2⋯Br1^ii^	0.93	2.70	3.554 (5)	153

Symmetry codes: (i) 
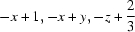
; (ii) 
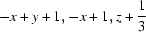
.

**Fig. 1.**
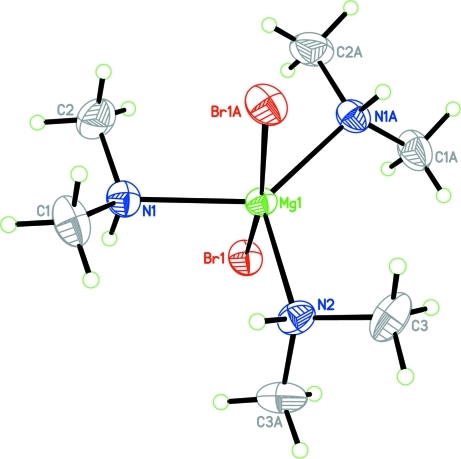
Perspective view of the title compound with the atom numbering scheme; displacement ellipsoids are at the 50% probability level; H atoms are drawn as small spheres of arbitrary radii. Only one of the two disordered moieties is shown.

## supplementary crystallographic information

### Comment

The solid-state structures of Mg—Br compounds feature coordination numbers of the Mg center from four as in [MgBr(Si*t*Bu_3_)(THF)]_2_ (Lerner *et al.*, 2003) to six as in [MgBr_2_(THF)_4_] (Lorbach *et al.*, 2007). Most of the Mg—Br compounds possess an octahedral coordination sphere which surrounds the Mg cation whereas only a few compounds are found in the Cambridge Structural Database with four- and five-coordinated Mg centers. We report here the X-ray crystal structure analysis of [MgBr_2_(NHMe_2_)_3_], the adduct of MgBr_2_ with three dimethylamine molecules, which was obtained as a by-product from the reaction of C_6_F_5_MgBr with BrB(NMe_2_) in Et_2_O.

### Experimental

At 273 K, BrB(NMe_2_) (3.5 g, 19.6 mmol) was added to a solution of C_6_F_5_MgBr in Et_2_O which was obtained from C_6_F_5_Br (4,6 g, 18.6 mmol) and Mg (0.5 g, 21.0 mmol) in 25 ml Et_2_O. After distillation of C_6_F_5_B(NMe_2_) (yield 73%) colourless crystals of the title compound were obtained as distillation residue.

### Refinement

H atoms were geometrically positioned and refined using a riding model with fixed individual displacement parameters [U_iso_(H) = 1.2 *U*_eq_(N) or U_iso_(H) = 1.5 *U*_eq_(C)] and with N—H = 0.93Å and C—H = 0.98 Å. The N atom and H atoms of one dimethylamine ligand are disordered over two equally occupied positions.

### Crystal data

**Table:**

[MgBr_2_(C_2_H_7_N)_3_]	*D*_x_ = 1.537 Mg m^−^^3^
*M**_r_* = 319.39	Mo *K*α radiation, λ = 0.71073 Å
Hexagonal, *P*3_2_21	Cell parameters from 5127 reflections
Hall symbol: P 32 2"	θ = 3.8–25.6°
*a* = 9.0951 (7) Å	µ = 5.88 mm^−^^1^
*c* = 14.4544 (12) Å	*T* = 173 K
*V* = 1035.49 (14) Å^3^	Block, colourless
*Z* = 3	0.25 × 0.25 × 0.23 mm
*F*(000) = 480	

### Data collection

**Table:**

Stoe IPDSII two-circle diffractometer	1285 independent reflections
Radiation source: fine-focus sealed tube	1223 reflections with *I* > 2σ(*I*)
graphite	*R*_int_ = 0.037
ω scans	θ_max_ = 25.6°, θ_min_ = 3.8°
Absorption correction: multi-scan (*MULABS*; Spek, 2003; Blessing, 1995)	*h* = −11→8
*T*_min_ = 0.321, *T*_max_ = 0.345	*k* = −7→11
5279 measured reflections	*l* = −17→17

### Refinement

**Table:**

Refinement on *F*^2^	Hydrogen site location: inferred from neighbouring sites
Least-squares matrix: full	H-atom parameters constrained
*R*[*F*^2^ > 2σ(*F*^2^)] = 0.022	*w* = 1/[σ^2^(*F*_o_^2^) + (0.0277*P*)^2^ + 0.2606*P*] where *P* = (*F*_o_^2^ + 2*F*_c_^2^)/3
*wR*(*F*^2^) = 0.049	(Δ/σ)_max_ = 0.001
*S* = 0.96	Δρ_max_ = 0.25 e Å^−^^3^
1285 reflections	Δρ_min_ = −0.33 e Å^−^^3^
61 parameters	Extinction correction: *SHELXL97* (Sheldrick, 2008), Fc^*^=kFc[1+0.001xFc^2^λ^3^/sin(2θ)]^-1/4^
0 restraints	Extinction coefficient: 0.0106 (8)
Primary atom site location: structure-invariant direct methods	Absolute structure: Flack (1983), 517 Friedel pairs
Secondary atom site location: difference Fourier map	Flack parameter: −0.012 (17)

### Special details

**Table:**

Geometry. All e.s.d.'s (except the e.s.d. in the dihedral angle between two l.s. planes) are estimated using the full covariance matrix. The cell e.s.d.'s are taken into account individually in the estimation of e.s.d.'s in distances, angles and torsion angles; correlations between e.s.d.'s in cell parameters are only used when they are defined by crystal symmetry. An approximate (isotropic) treatment of cell e.s.d.'s is used for estimating e.s.d.'s involving l.s. planes.
Refinement. Refinement of *F*^2^ against ALL reflections. The weighted *R*-factor *wR* and goodness of fit *S* are based on *F*^2^, conventional *R*-factors *R* are based on *F*, with *F* set to zero for negative *F*^2^. The threshold expression of *F*^2^ > σ(*F*^2^) is used only for calculating *R*-factors(gt) *etc*. and is not relevant to the choice of reflections for refinement. *R*-factors based on *F*^2^ are statistically about twice as large as those based on *F*, and *R*- factors based on ALL data will be even larger.

### Fractional atomic coordinates and isotropic or equivalent isotropic displacement parameters (Å^2^)

**Table:**

	*x*	*y*	*z*	*U*_iso_*/*U*_eq_	Occ. (<1)
Br1	0.39287 (4)	0.37977 (4)	0.318621 (17)	0.03405 (12)	
Mg1	0.41479 (13)	0.41479 (13)	0.5000	0.0231 (3)	
N1	0.4463 (3)	0.1927 (3)	0.50042 (15)	0.0301 (5)	
H1	0.4887	0.1897	0.4423	0.036*	
C1	0.5661 (5)	0.1872 (5)	0.5683 (2)	0.0441 (9)	
H1A	0.6768	0.2910	0.5630	0.066*	
H1B	0.5789	0.0883	0.5556	0.066*	
H1C	0.5217	0.1787	0.6310	0.066*	
C2	0.2791 (5)	0.0368 (4)	0.5070 (3)	0.0474 (9)	
H2A	0.2012	0.0419	0.4620	0.071*	
H2B	0.2334	0.0270	0.5695	0.071*	
H2C	0.2916	−0.0621	0.4939	0.071*	
N2	0.6685 (6)	0.6265 (6)	0.5188 (3)	0.0293 (12)	0.50
H2	0.7183	0.5896	0.5623	0.035*	0.50
C3	0.6750 (5)	0.7809 (4)	0.5616 (2)	0.0444 (8)	
H3A	0.7933	0.8716	0.5684	0.067*	0.50
H3B	0.6206	0.7508	0.6226	0.067*	0.50
H3C	0.6143	0.8201	0.5218	0.067*	0.50
H3D	0.7715	0.8920	0.5457	0.067*	0.50
H3E	0.5785	0.7940	0.5803	0.067*	0.50
H3F	0.7063	0.7309	0.6125	0.067*	0.50

### Atomic displacement parameters (Å^2^)

**Table:**

	*U*^11^	*U*^22^	*U*^33^	*U*^12^	*U*^13^	*U*^23^
Br1	0.0453 (2)	0.04152 (19)	0.02015 (14)	0.02533 (16)	0.00289 (12)	−0.00057 (12)
Mg1	0.0233 (5)	0.0233 (5)	0.0200 (5)	0.0096 (6)	0.0012 (2)	−0.0012 (2)
N1	0.0368 (15)	0.0336 (14)	0.0232 (12)	0.0199 (12)	0.0007 (12)	−0.0026 (11)
C1	0.055 (2)	0.057 (2)	0.0385 (17)	0.041 (2)	−0.0087 (17)	−0.0075 (17)
C2	0.048 (2)	0.0276 (17)	0.057 (2)	0.0121 (16)	0.0043 (18)	−0.0044 (15)
N2	0.027 (2)	0.027 (3)	0.028 (3)	0.010 (2)	−0.0027 (19)	0.005 (2)
C3	0.054 (2)	0.0249 (16)	0.0425 (18)	0.0111 (16)	−0.0098 (17)	−0.0037 (14)

### Geometric parameters (Å, °)

**Table:**

Br1—Mg1	2.6365 (4)	C2—H2B	0.9800
Mg1—N2	2.159 (5)	C2—H2C	0.9800
Mg1—N2^i^	2.159 (5)	N2—N2^i^	0.856 (9)
Mg1—N1	2.177 (3)	N2—C3^i^	1.463 (6)
Mg1—N1^i^	2.177 (3)	N2—C3	1.508 (6)
Mg1—Br1^i^	2.6365 (4)	N2—H2	0.9300
N1—C2	1.475 (4)	C3—N2^i^	1.463 (6)
N1—C1	1.486 (4)	C3—H3A	0.9800
N1—H1	0.9300	C3—H3B	0.9800
C1—H1A	0.9800	C3—H3C	0.9800
C1—H1B	0.9800	C3—H3D	0.9788
C1—H1C	0.9800	C3—H3E	0.9803
C2—H2A	0.9800	C3—H3F	0.9780
			
N2—Mg1—N2^i^	22.9 (2)	C3^i^—N2—C3	110.5 (4)
N2—Mg1—N1	104.45 (16)	N2^i^—N2—Mg1	78.57 (12)
N2^i^—Mg1—N1	122.07 (17)	C3^i^—N2—Mg1	116.3 (3)
N2—Mg1—N1^i^	122.07 (17)	C3—N2—Mg1	114.1 (3)
N2^i^—Mg1—N1^i^	104.45 (16)	N2^i^—N2—H2	175.2
N1—Mg1—N1^i^	133.08 (16)	C3^i^—N2—H2	104.6
N2—Mg1—Br1^i^	88.69 (13)	C3—N2—H2	105.1
N2^i^—Mg1—Br1^i^	102.44 (14)	Mg1—N2—H2	105.0
N1—Mg1—Br1^i^	89.66 (6)	N2^i^—C3—H3A	119.2
N1^i^—Mg1—Br1^i^	85.86 (6)	N2—C3—H3A	109.9
N2—Mg1—Br1	102.44 (14)	N2^i^—C3—H3B	125.9
N2^i^—Mg1—Br1	88.69 (13)	N2—C3—H3B	109.2
N1—Mg1—Br1	85.86 (6)	H3A—C3—H3B	109.5
N1^i^—Mg1—Br1	89.66 (6)	N2^i^—C3—H3C	76.1
Br1^i^—Mg1—Br1	168.72 (5)	N2—C3—H3C	109.3
C2—N1—C1	110.2 (3)	H3A—C3—H3C	109.5
C2—N1—Mg1	110.0 (2)	H3B—C3—H3C	109.5
C1—N1—Mg1	118.2 (2)	N2^i^—C3—H3D	109.6
C2—N1—H1	105.9	N2—C3—H3D	117.5
C1—N1—H1	105.9	H3B—C3—H3D	124.5
Mg1—N1—H1	105.9	H3C—C3—H3D	82.1
N1—C1—H1A	109.5	N2^i^—C3—H3E	109.2
N1—C1—H1B	109.5	N2—C3—H3E	127.2
H1A—C1—H1B	109.5	H3A—C3—H3E	122.9
N1—C1—H1C	109.5	H3B—C3—H3E	55.2
H1A—C1—H1C	109.5	H3C—C3—H3E	54.3
H1B—C1—H1C	109.5	H3D—C3—H3E	109.5
N1—C2—H2A	109.5	N2^i^—C3—H3F	109.2
N1—C2—H2B	109.5	N2—C3—H3F	76.2
H2A—C2—H2B	109.5	H3A—C3—H3F	82.1
N1—C2—H2C	109.5	H3B—C3—H3F	54.4
H2A—C2—H2C	109.5	H3C—C3—H3F	163.4
H2B—C2—H2C	109.5	H3D—C3—H3F	109.7
N2^i^—N2—C3^i^	76.2 (6)	H3E—C3—H3F	109.6
N2^i^—N2—C3	70.4 (6)		
			
N2—Mg1—N1—C2	168.6 (2)	Br1—Mg1—N2—N2^i^	54.3 (8)
N2^i^—Mg1—N1—C2	−175.4 (2)	N2^i^—Mg1—N2—C3^i^	−68.2 (7)
N1^i^—Mg1—N1—C2	−3.95 (19)	N1—Mg1—N2—C3^i^	75.0 (3)
Br1^i^—Mg1—N1—C2	80.0 (2)	N1^i^—Mg1—N2—C3^i^	−111.4 (3)
Br1—Mg1—N1—C2	−89.6 (2)	Br1^i^—Mg1—N2—C3^i^	164.3 (3)
N2—Mg1—N1—C1	40.9 (3)	Br1—Mg1—N2—C3^i^	−13.9 (4)
N2^i^—Mg1—N1—C1	56.8 (3)	N2^i^—Mg1—N2—C3	62.2 (7)
N1^i^—Mg1—N1—C1	−131.7 (2)	N1—Mg1—N2—C3	−154.6 (3)
Br1^i^—Mg1—N1—C1	−47.7 (2)	N1^i^—Mg1—N2—C3	19.0 (4)
Br1—Mg1—N1—C1	142.7 (2)	Br1^i^—Mg1—N2—C3	−65.3 (3)
N1—Mg1—N2—N2^i^	143.2 (7)	Br1—Mg1—N2—C3	116.5 (3)
N1^i^—Mg1—N2—N2^i^	−43.2 (8)	C3^i^—N2—C3—N2^i^	66.2 (5)
Br1^i^—Mg1—N2—N2^i^	−127.5 (7)	Mg1—N2—C3—N2^i^	−67.0 (4)

Symmetry codes: (i) *y*, *x*, −*z*+1.

### Hydrogen-bond geometry (Å, °)

**Table:**

*D*—H···*A*	*D*—H	H···*A*	*D*···*A*	*D*—H···*A*
N1—H1···Br1^ii^	0.93	2.90	3.638 (2)	137
N2—H2···Br1^iii^	0.93	2.70	3.554 (5)	153

Symmetry codes: (ii) −*x*+1, −*x*+*y*, −*z*+2/3; (iii) −*x*+*y*+1, −*x*+1, *z*+1/3.
